# Carcinoembryonic antigen-targeted photodynamic therapy in colorectal cancer models

**DOI:** 10.1186/s13550-019-0580-z

**Published:** 2019-12-11

**Authors:** Fortuné M. K. Elekonawo, Desirée L. Bos, David M. Goldenberg, Otto C. Boerman, Mark Rijpkema

**Affiliations:** 1 Department of Radiology and Nuclear Medicine, Radboud University Medical Center, PO Box 9101, 6500 HB Nijmegen, The Netherlands; 2 Center for Molecular Medicine and Immunology, Mendham, NJ USA; 3 Immunomedics, Inc. and IBC Pharmaceuticals, Inc., Morris Plains, NJ USA

**Keywords:** Colorectal cancer, Targeted photodynamic therapy, Carcinoembryonic antigen, Targeted, IRDye700DX

## Abstract

**Background:**

In colorectal cancer, survival of patients is drastically reduced when complete resection is hampered by involvement of critical structures. Targeted photodynamic therapy (tPDT) is a local and targeted therapy which could play a role in eradicating residual tumor cells after incomplete resection. Since carcinoembryonic antigen (CEA; CEACAM5) is abundantly overexpressed in colorectal cancer, it is a potential target for tPDT of colorectal cancer.

**Methods:**

To address the potential of CEA-targeted PDT, we compared colorectal cancer cell lines with different CEA-expression levels (SW-48, SW-480, SW-620, SW-1222, WiDr, HT-29, DLD-1, LS174T, and LoVo) under identical experimental conditions. We evaluated the susceptibility to tPDT by varying radiant exposure and concentration of our antibody conjugate (DTPA-hMN-14-IRDye700DX). Finally, we assessed the efficacy of tPDT in vivo in 18 mice (BALB/cAnNRj-*Foxn1*^*nu/nu*^) with subcutaneously xenografted LoVo tumors.

**Results:**

In vitro, the treatment effect of tPDT varied per cell line and was dependent on both radiant exposure and antibody concentration. Under standardized conditions (94.5 J/cm^2^ and 0.5 μg/μL antibody conjugate concentration), the effect of tPDT was higher in cells with higher CEA availability: SW-1222, LS174T, LoVo, and SW-48 (22.8%, 52.8%, 49.9%, and 51.9% reduction of viable cells, respectively) compared to cells with lower CEA availability. Compared to control groups (light or antibody conjugate only), tumor growth rate was reduced in mice with s.c. LoVo tumors receiving tPDT.

**Conclusion:**

Our findings suggest cells (and tumors) have different levels of susceptibility for tPDT even though they all express CEA. Furthermore, tPDT can effectively reduce tumor growth in vivo.

## Introduction

Surgery is a cornerstone in curative treatment of colorectal cancer; however, incomplete resection can drastically reduce survival after surgery [1, 2]. Radical resection of tumors might be hampered by involvement of critical structures including large vessels, nerves, or visceral organs. In advanced-stage cancers, adjuvant local or systemic therapies can improve survival after (incomplete) resection. Survival of systemically treated patients with advanced colorectal cancer has improved with modern systemic therapies [3], but the maximum dose of systemic therapy is limited by toxicity and side effects [4].

Photodynamic therapy (PDT) could play a role in overcoming the limitations of incomplete radical resection and toxicity from systemic therapy. PDT has gained a role in treatment in different fields of oncology nowadays [5–8]. It can be applied as standalone treatment modality, although it might also serve as adjuvant treatment to surgery after incomplete resection [9].

The principle of PDT is based on combining three non-toxic components: oxygen, light, and a photosensitizer. The photosensitizer is excited with the physical energy of non-ionizing light (of a specific wavelength) which, through a series of photochemical reactions, results in formation of highly reactive oxygen species (ROS) [10, 11]. In turn, ROS may induce local cell apoptosis and necrosis and/or cause microvascular damage. Furthermore, a change in photosensitizer structure and hydrophilicity has been proposed to contribute to cell damage [12]. Preferably, accumulation of the photosensitizer should be tumor-specific to prevent extensive damage to the normal tissue surrounding the tumor and to increase the intratumoral dose. Therefore, a tumor-targeted PDT approach has been developed [13]. In targeted photodynamic therapy (tPDT), a tissue of interest is selectively localized using a targeting vehicle conjugated to a photosensitizer. When the photosensitizer-vehicle conjugate has accumulated in targeted tissue, light of a specific wavelength is administered locally, making this therapy highly specific.

Carcinoembryonic antigen (CEA) is a membrane-anchored glycoprotein and is overexpressed in 90–95% of colorectal cancer cases. Therefore, CEA can be used for primary targeting of colorectal carcinomas. hMN-14 (labetuzumab) is an IgG directed against the carcinoembryonic antigen-related cell adhesion molecule 5 with high affinity [14]. However, different tumors express different amounts of CEA. Therefore, in clinical practice CEA-targeted PDT might only be useful in tumors with sufficient CEA expression, when complete tumor resection is hampered by the presence of critical structures that are to be preserved.

Here, we investigate whether the effect of tumor-targeted PDT is influenced by the availability of CEA on the cell surface of tumor cells with different CEA expression levels. Our multimodal conjugate (DTPA-hMN-14-700DX) consists of the humanized anti-CEA antibody, hMN-14 (labetuzumab), the photosensitizer IRDye700DX, and the chelator diethylenetriaminepentaacetic acid (DTPA). DTPA allows radiolabeling with ^111^In and subsequent in vivo tumor detection and precise quantification of the antibody conjugate accumulation. After the in vitro experiments on different colorectal cancer cell lines, the therapeutic effect of tPDT was evaluated in vivo in a xenograft mouse model.

## Materials and methods

### Cell culture

CEA-expressing human colon adenocarcinoma (primary or metastatic) cell lines were obtained from the American Type Culture Collection (ATCC, Manassas, VA, USA). SW-1222 cells were obtained from Sigma-Aldrich (Saint Louis, MO, USA). LS174T, SW-620, SW-480, SW-48, DLD-1, and HT-29 were cultured in RPMI-1640 (Gibco^TM^, Dun Laoghaire, Ireland) supplemented with 10% fetal bovine serum (FBS) and 2 mM L-glutamine. WiDr and SW-1222 were cultured in DMEM high glucose (Gibco) supplemented with 10% FBS. LoVo was cultured in Ham’s F-12 Nutrient mix GLUTAMAX (Gibco) supplemented with 10% FBS. No antibiotic additives were used. All cells tested negative for Mycoplasma. Cells were cultured in tissue culture flasks in a humidified incubator at 37 °C in an atmosphere of 95% air and 5% CO_2_. Cells were harvested with 0.05% trypsin-EDTA (ethylenediaminetetraacetic acid).

### DTPA-hMN-14-IRDye700DX conjugation

hMN-14 (labetuzumab) was kindly provided by Immunomedics, Inc., Morris Plains, NJ, USA. It was conjugated with IRDye700DX-NHS (LI-COR, Lincoln, NE, USA) and SCN-Bz-dieethylenetriaminepentaacetic acid (DTPA) (Macrocyclics, Plano, TX, USA) in two steps. First, hMN-14 was conjugated with IRDye700DX-NHS in 0.1 M NaHCO_3_, pH 8.5, with a 10-fold molar excess of IRDye700DX-NHS. Next, the reaction mixture was incubated for 1 h at room temperature on an orbital shaker and protected from light. Second, SCN-Bz-DTPA in 0.1 M NaHCO_3_, pH 9.5 was added to the reaction mixture in a 10-fold molar excess. After another hour of incubation on the orbital shaker in the dark, the mixture was dialyzed in a Slide-A-Lyzer (10 kDa cutoff; Thermo Fisher Scientific, Waltham, MA, USA) against phosphate-buffered saline (PBS) containing 2 g/L Chelex® 100 Resin (Bio-Rad Laboratories, Inc.; Hercules, CA, USA). The final concentration of the conjugate was determined spectrophotometrically at 280 nm (Ultrospec 2000 spectrophotometer; Pharmacia Biotech), correcting for the absorption of IRDye700DX at that wavelength (3%, according to the manufacturer’s protocol). The molar substitution ratio of IRDye700DX was determined spectrophotometrically at 648 nm and reached 4.5.

### Radiolabeling of the hMN-14 conjugate

Briefly, [^111^In]InCl_3_ (Curium, Petten, The Netherlands) was added to DTPA-hMN-14-IRDye700DX in 3 V of 0.1 M 2-(N-morpholino)ethanesulfonic acid (MES), pH 5.5. After 30 min of incubation at room temperature, 50 mM EDTA was added to the labeling reaction to a final concentration of 5 mM to chelate unincorporated [^111^In]InCl_3_. Labeling efficiency was determined by instant thin-layer chromatography on Varian silicagel strips (ITLC-SG; Agilent Technologies, Amstelveen, The Netherlands) using 0.1 mM ammonium acetate (NH_4_Ac) buffer with 0.1 M EDTA, pH 5.5 as the mobile phase and labeling efficiency reached > 95%.

For the in vitro binding assay, DTPA-hMN-14-IRDye700DX was radiolabeled with 0.5 MBq/μg of [^111^In]InCl_3_. For the biodistribution studies, in two mice with s.c. LoVo tumors, DTPA-hMN-14-IRDye700DX was radiolabeled with 0.23 MBq/μg of [^111^In]InCl_3_.

### In vitro binding assay

The radiolabeled conjugate was diluted to contain 2.4 kBq per μL. To count the amount of activity, we used a shielded 3”-well-type γ-counter (Perkin-Elmer, Boston, MA, USA). Cells were counted and placed in culture medium supplemented with 0.5% Bovine Serum Albumin (BSA; Sigma-Aldrich Chemie N.V., Zwijndrecht, Netherlands) (Binding buffer; BB) at a concentration of 1 × 10^7^ cells per milligram. Next, 4 × 10^6^ cells were pipetted in a 1.5 mL eppendorf tube (2 tubes per cell line; experiment performed in triplicate). We added 240 kBq of our radiolabeled antibody conjugate to cells in each tube. An excess of unlabeled DTPA-hMN-14- IRDye700DX (1.9 μg) was added to the last three tubes of each cell lines to determine the amount of nonspecific binding of the conjugate.

Cells were incubated at 37 °C with 5% CO_2_ for 4 h. Following incubation, cells were centrifuged at 805 g for 5 min, the supernatant was removed and the remaining activity in the tubes was measured in a γ-counter. A 100-μL standard, representing 100% activity, was measured in triplicate simultaneously. The antibody binding in the presence of an excess unlabeled antibody was subtracted from the antibody conjugate binding for each cell line. This results in a measure for the specific binding of our antibody conjugate, expressed as percentage of the total amount of added antibody conjugate.

### PDT device

We used a standardized 690 nm (SMBB690D-1100-02) high-output LED (Marubeni America Corporation; Santa Clara, CA, USA) device for illumination of targets [15]. All experiments were performed with the device set to its maximum power output of 200 mW/cm^2^. To adjust light fluency rate, we varied the distance between light source and target. The bottom of the in vitro setup was transparent to prevent excessive heat generation and light reflection from the surface beneath the treated cells.

### In vitro photodynamic therapy

**Fig. 1:**
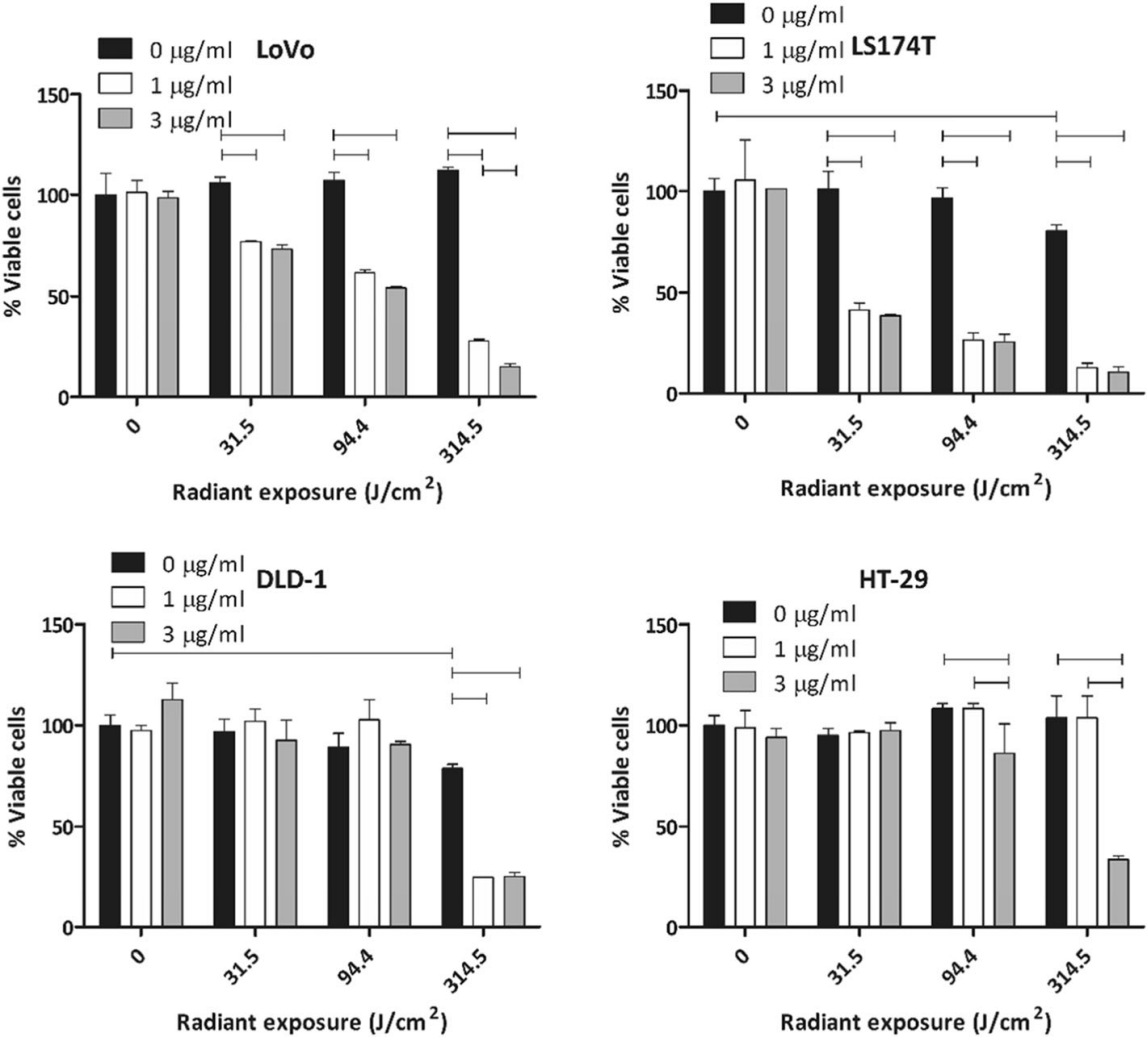
In vitro cell viability (tPDT effect) on LoVo, LS174T, DLD-1, and HT-29 cells treated with different doses of light and antibody conjugate concentration. Statistically significant differences (*p* < 0.05) are indicated with horizontal bars

To gain insight into the effects of tPDT and CEA availability, LoVo, LS174T, DLD-1, and HT-29 cells were treated with different antibody conjugate doses and light doses (Fig. 1). Next, to directly compare the therapeutic effect on the 9 cell lines, all cells were treated with the same light and antibody-conjugate dose. Briefly, 2 × 10^5^ cells per well were plated in 2 wells of a 24-well plate (experiment performed in triplicate) 1 day prior to treatment. On the day of treatment, 0.5 μg/μL DTPA-hMN-14-700DX was added in 500 μL of BB. In this experiment, tPDT was performed with a radiant exposure of 94.5 J/cm^2^.

For both in vitro experiments, after harvesting, cells were plated in transparent 24-well plates (Corning Inc.; Corning, NY, USA) (1.5–2.0·10^5^ cells per well) and allowed to adhere overnight. After cells were adherent, they were incubated with the antibody-conjugate in BB for 4 h. Following incubation, BB was removed and cells were washed once with phosphate-buffered saline to remove unbound antibody. Next, fresh medium was added and cells were exposed to specific radiant exposures. Cells rested for 1 h after light exposure. Next, cells were washed to remove cell debris. Hereafter, cell viability was assessed using the cell titer Glo® (Promega Corporation; Madison, WI, USA) luminescent cell viability assay. Incubation in demineralized water was used as positive control for 100% cell death.

### Animals

All animal experiments were approved by the Dutch Central Committee for Animal Experiments, and local protocols were approved by the Institutional Animal Welfare Committee of the Radboud University Medical Center and were conducted in accordance to the guidelines of the Revised Dutch Act on Animal Experimentation (2014).

Twenty male BALB/cAnNRj-*Foxn1*^*nu/nu*^ nude mice (7 to 9 weeks old, 18–22 g body weight; Janvier labs; Le Genest-Saint-Isle, France) were housed in individually ventilated cages (5 mice per cage) under standard non-sterile conditions. Mice had free access to standard animal chow and water. Animals were adapted to laboratory conditions for 1 week before experimental use. Subcutaneous (s.c.) tumors were induced by s.c. injection of 5 × 10^6^ freshly harvested LoVo cells. Tumors grew in all mice that were injected s.c.

### In vivo targeted photodynamic therapy

When average tumor size reached 45 mm^3^ and after stratification based on tumor size, 18 mice were randomly allocated into three experimental groups (treatment tPDT, PBS + 0.5% BSA with light exposure, antibody-conjugate without light exposure), 6 mice per group), mice were injected with 30 μg of unlabeled DTPA-hMN-14-IRDye700DX or PBS + 0.5% BSA via a 200-μl tail vein injection.

Tumors of mice in one control group (*n* = 6) and the treatment group (*n* = 6) were selectively exposed to 300 J/cm^2^ of near infrared (NIR) light under inhalation anesthesia (2.5% isoflurane mixed with 100% O_2_ (1 L/min)). All mice, including non-irradiated controls, were anesthetized for 12 min. The liver and other organs were protected from exposure to the NIR light by covering those areas with a gauze and aluminum foil.

Treatment efficacy was determined based on tumor growth. Tumor diameters were measured in three dimensions by a blinded observer using a caliper three times per week. Tumor volume was calculated as the volume of an ellipsoid: 4/3 ^.^ *π* ^.^ *r*1 ∙ *r*2 ∙ *r*3. Herein, *r* was calculated by dividing the tumor length, width, or height by two. Mice were euthanized by O_2_/CO_2_ asphyxiation when tumor volume exceeded more than 1000 mm^3^. One mouse in the treatment group was excluded from the analyses, since we failed to irradiate its tumor with light (the aluminum foil shifted and covered the tumor).

### Biodistribution

Two mice were used to determine the biodistribution of ^111^In-labeled DTPA-hMN-14-IRDye700DX (Additional file 1: Figure S2) ex vivo. Twenty-four hours after injection of the tracer, mice were euthanized and tissues of interest (tumor, muscle, lung, spleen, kidney, liver, pancreas, stomach, and duodenum) were dissected and weighed after which activity was measured in the γ-counter. Blood samples were obtained by cardiac puncture. For calculation of the uptake of activity in each tissue as a fraction of the injected activity, three aliquots of the injection dose were counted in the γ-counter simultaneously.

### Statistical analyses

Statistical analyses were performed using GraphPad Prism version 5.03 (GraphPad Software, Inc.; San Diego, CA, USA) and Statistical Package for Social Sciences, Version 22.0 (IBM Corp.; Armonk, NY, USA). A two-way ANOVA with Bonferroni correction for multiple testing was performed to analyze the effects of radiant exposure and antibody dose in the in vitro experiment with 9 different colorectal cancer cell lines. Furthermore, a one-way ANOVA with Dunnet correction for multiple testing was performed to compare the control condition without antibody and light (absolute control) with the control condition with light (intern control). For the in vivo experiment, a one-way ANOVA with Bonferroni correction for multiple testing was performed. A *p* value < 0.05 was used to reject the null hypothesis. Data are presented as mean and standard deviation.

## Results

First, we assessed the binding capacity of ^111^In-DTPA-hMN-14-IRdye700DX to cell lines with different CEA expression levels. This capacity can be considered as a surrogate value for accessible CEA epitopes on the cell surface. Additional file 2: Figure S1 illustrates that the cells used in this study can be roughly classified into two groups: low and high CEA accessibility.

To gain insight into the effects of tPDT, we treated 2 cell lines with low CEA availability and 2 cell lines with high CEA availability under different conditions, varying both the antibody conjugate concentration and radiant exposure (light dose).

LS174T and LoVo cells (both high CEA availability) showed a light dose-dependent increase of therapeutic efficacy of tPDT, regardless of antibody conjugate concentration (Fig. 1). At the highest light dose (314.5 J/cm^2^), the effect of tPDT on LoVo was higher at 3 μg/mL compared to 1 μg/mL antibody conjugate concentration (27.8% vs 15.1% viable cells, *p* = 0.002). At lower light doses, we did not observe an additional effect of increasing the antibody conjugate dose. Similar to LoVo, in LS174T the effect of tPDT increased with increasing light dose (Fig. 1). So, in the cell lines with higher CEA availability, we observed a clear effect of increasing light dose and antibody conjugate dose on the effect of tPDT. In contrast, in cell lines with lower CEA availability (DLD-1 and HT-29), we did not observe this variation in susceptibility for tPDT. Decreased cell viability due to tPDT in DLD-1 was only observed at the highest dose level of light (*p* < 0.001) and was not dependent on the antibody conjugate concentration used. In HT-29 cells (low to moderate CEA availability), we did not find an effect of tPDT at 1 μg/mL of antibody conjugate. At 3 μg/mL, however, there was an effect of tPDT at the two highest light doses (94.4 and 314.5 J/cm^2^). From these experiments, we concluded that the effect of antibody conjugate dose and light dose on the efficacy of tPDT seem to be dependent on the CEA availability in tumor cells.

**Fig. 2:**
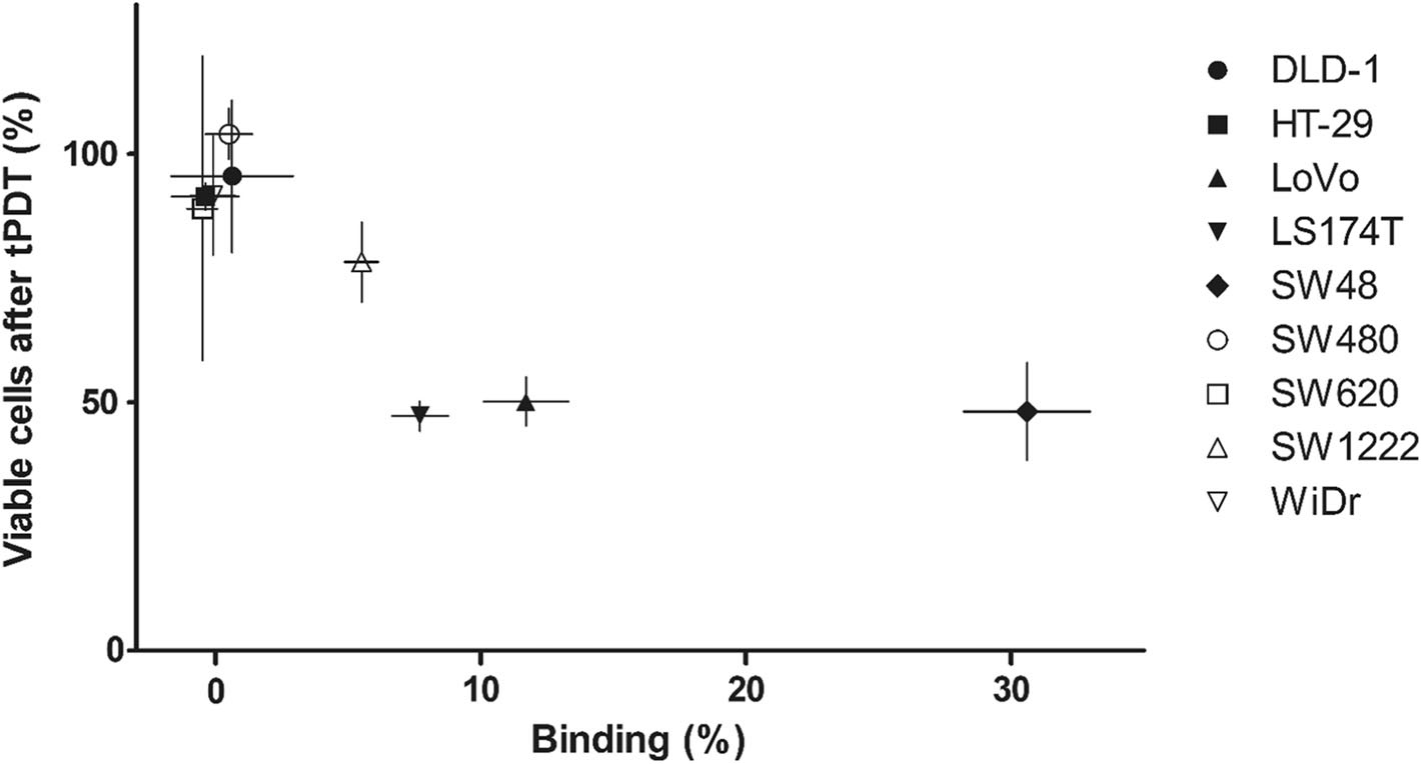
Relation between the tPDT effect (amount of remaining viable cells 1 h after treatment) and antibody conjugate binding (expressed as percentage of the total amount of added antibody conjugate) for the nine different cell lines. Note that in general more binding (more accessible/targetable CEA sites) leads to a greater effect of tPDT (fewer viable cells)

Subsequently, we performed a separate experiment to investigate the relationship between CEA availability and tPDT effects. To explore this relationship, we included 9 cell lines with varying CEA expression levels. Figure 2 summarizes our findings on tPDT effect and its relation to amount of antibody conjugate binding of the different cell lines. No treatment effect was observed in cells with less than 5% specific binding of the antibody conjugate, which represents low CEA availability (HT-29, SW620, SW480, WiDr, and DLD-1). SW-1222 (5.5% specific binding) showed a moderate effect of tPDT after treatment (78.2% viable cells). LS174T, LoVo, and SW48 (7.7, 11.7, and 30.6% specific binding, respectively) were more sensitive to treatment with tPDT (47.2%, 50.1%, and 48.1% viable cells after treatment, respectively), suggesting that a minimum level of antibody conjugate binding to the cells is required for effective treatment with tPDT.

**Fig. 3:**
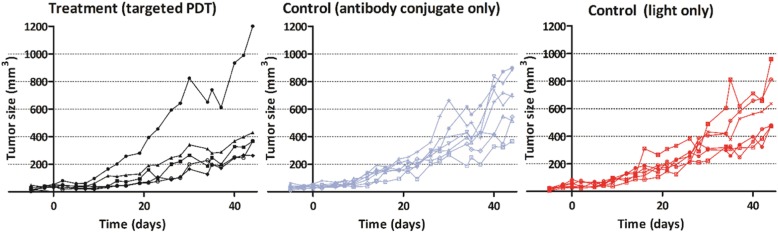
In vivo tumor growth progression after targeted PDT treatment (day 0) and control conditions followed up three times per week. The two control groups: DTPA-hMN-14-IRDye700DX only group (without light exposure) in blue and the light-only group in red. Only 5 mice were treated due to a technical incident during the treatment of the 6th mouse. In general, mice in the tPDT (treatment) group show a delayed tumor growth pattern. One of the treated mice had a more aggressive growth pattern

Subsequently, we evaluated the ability of tPDT to reduce in vivo tumor progression in a xenograft mouse model. Before evaluating the treatment, we performed an ex vivo biodistribution of ^111^In-labeled DTPA-hMN-14-IRDye700DX. We found 13.7 ± 3.8 %ID/g of our tracer in tumor, 8.8 ± 0.5 %ID/g in spleen and 29.4 ± 2.6 %ID/g in liver tissue (Additional file 2: Figure S1). In mice treated with tPDT, we generally observed slower tumor growth than the control groups (*n* = 17, *p* > 0.05). The tumor of one mouse in the treatment group, however, showed a more aggressive growth pattern after treatment (mouse 1, Fig. 3). This was possibly caused by incomplete tumor irradiation during tPDT, but because this was not reported during treatment of this mouse, this dataset was not rejected from the analyses.

## Discussion

The current study demonstrates that in vitro tumor cell lines with different levels of CEA targetability have varying susceptibility to tPDT. Furthermore, the amount of ^111^In-DTPA-hMN-14-IRDye700DX binding to these cells seems to play a crucial role in the efficacy of tPDT. Our findings suggest that only cell lines that can be targeted with higher amounts of antibody-conjugate can be treated effectively with tPDT. In vivo, we observed a trend towards tumor growth delay in xenografted tumors of tPDT-treated mice.

In the tested cell lines, higher light dose increased the efficacy of tPDT. However, in DLD-1 and LS174T cells, the maximum light dose led to a statistically significant decrease in cell viability even without the presence of the antibody conjugate (Fig. 1). This suggests that the energy provided by the light source may have been excessive in this condition. Hyperthermia can lead to decreased cell survival in (monolayer) cells and is likely to be responsible for the decrease in cell viability of the 0 μg/mL control condition of LS174T and DLD-1 [16, 17].

In this study, we included a large series of CEA-expressing cell lines in a direct comparison. To compare the tPDT effect in all cell lines under identical conditions, we performed a single experiment with fixed conditions: 0.5 μg/μL ^111^In-DTPA-hMN-14-IRDye700DX and total radiant exposure 94.5 J/cm^2^. Higher radiant exposures could have an additional thermal effect on several cell lines (see above). Since we chose 94.5 J/cm^2^, the observed effects depicted in Fig. 2 are considered to be only tPDT-mediated, but may be an underestimation of the maximum tPDT effect that could be achieved. More specific binding of ^111^In-DTPA-hMN-14-IRDye700DX resulted in a larger treatment efficacy of tPDT in vitro (Fig. 2). These findings are consistent with previous studies that showed an increased efficacy of tPDT when there is more photosensitizer available in the tumor [13].

Increasing the number of photosensitizer moieties per antibody molecule (substitution ratio) might further increase the amount of photosensitizer in tumors. However, as we use a random conjugation method, excessive conjugation of the photosensitizer to an antibody might affect the antibody binding affinity. Additionally, when conjugating high amounts of a photosensitizer or in general a fluorescent dye to an antibody, changes in chemical properties (lipophilicity and net charge) may lead to faster blood clearance, less tumor uptake, and more accumulation in liver and spleen [18–20]. Findings from these studies are in line with the findings of the biodistribution performed in the current study. Random conjugation strategies can, therefore, be considered a limitation regarding the preparation of an antibody-photosensitizer conjugate. Our results also suggest that tPDT efficacy is dependent on a combination of radiant exposure and amount of photosensitizer that is present on the target. In addition, different coping strategies of tumor cells to tPDT-induced damage may affect treatment efficacy, but this was not taken into account in the current study.

In contrast to in vitro tPDT, in vivo tPDT can be particularly challenging due to the multifactorial nature of the treatment and treatment effect. In the current study, we tested the efficacy of tPDT with subcutaneous LoVo tumors in BALB/c nude mice. LoVo cells were chosen as a representative because the antibody-conjugate binding to the cells was the most representative of the 3 cell lines with the a highest binding percentage (SW-48, LoVo, and LS174T) (Additional file 2: Figure S1). We observed a steady increase in tumor size in the control groups during 6 weeks. The treated mice showed a slower tumor growth pattern in general (Fig. 3). The delay in tumor growth was, however, not statistically different compared to the control groups. This was caused by one treated mouse that showed an accelerated tumor growth pattern. When cells survive after (incomplete) tPDT, cell cycle progression might be stimulated, resulting in increased proliferation, and invasive and metastatic growth [21]. This can particularly occur when treatment is not adequate. Furthermore, in a single tumor, heterogeneity in CEA expression may exist, which could be a limitation in adequate tumor targeting [22, 23]. Earlier studies indicate that host immunity can also play a crucial role in achieving a successful effect of (targeted) PDT [10, 24].

However, due to the use of an immunodeficient mouse model, the effects of immune system involvement could not be assessed in the current study. Moreover, fractionated and repeated tPDT might enhance the tPDT effect, as observed earlier by Mitsunaga and colleagues [25].

During the past years, targeted PDT approaches have gained more clinical and scientific interest. In the clinical situation, tPDT may mainly have a role as adjuvant therapy to surgery [9]. The main advantage of tPDT over other adjuvant therapies is the ability to selectively apply therapy to local areas and tissues of interest, thereby minimizing side effects. When combined with NIR fluorescence or nuclear imaging, its potential for clinical use as theranostic approach is highlighted even further [26, 27].

Here, we focused on tPDT in a colorectal cancer model using a humanized monoclonal antibody directed against CEA (CEACAM5). Since other primary epithelial malignancies also express varying levels of CEA, CEA-targeted PDT could potentially be used in treatment of other malignancies as well. These include carcinomas of the gall bladder, urinary bladder, stomach, pancreas, ovary, endometrium, and lung [28]. Other conjugates of the same antibody have already been used in (clinical) studies investigating fluorescence and radioguided surgery, radioimmunotherapy, and antibody-drug conjugates [29–35].

Our findings are in line with work by Shirasu et al., who showed phototoxic and dose-dependent effects of CEA-targeted PDT, with their anti-CEA antibody C2-45 conjugated with IRDye700DX [36]. Our radiolabeled antibody conjugate, ^111^In-DTPA-hMN-14-IRDye700DX, allows precise quantification of the tracer both in vivo and in vitro, and may enable SPECT or PET imaging prior to surgery. During surgery, when tumors are covered by overlying tissue, the radiolabel could guide the surgeon to the area of interest, using a gamma probe prior to resection of tumors [29, 37]. Subsequently, tumor margins may be visualized using near-infrared fluorescence imaging using the same radiolabeled antibody conjugate. Lastly, the photosensitizer can be irradiated to treat residual tumor that was excluded from excision [9].

## Conclusions

Our findings suggest cells (and tumors) have different levels of susceptibility for tPDT even though they all express CEA. Furthermore, tPDT can effectively reduce tumor growth in vivo. In the current study, we treated whole subcutaneous xenografted tumors, whereas in the clinical situation one would optimally use tPDT after resection to treat (microscopically) small amounts of residual tumor cells. Thus, our findings could even be an underestimation of clinically achievable results with tPDT.

## Supplementary information

**Additional file 1: Figure S2**. Ex vivo biodistribution of two mice with subcutaneous LoVo tumors, one day after ^111^In-labeled DTPA-hMN-14-IRDye700DX injection.

**Additional file 2: Figure S1**. the CEA binding availability of the cell lines used in this study. Note the (arbitrary) classification in high (>5% specific binding) and low (<5% specific binding) binding of DTPA-hMN-14-IRDye700DX.

## Data Availability

The datasets used and/or analyzed during the current study are available from the corresponding author on reasonable request.

## Abbreviations

BB: Binding buffer
CEA: Carcinoembryonic antigen
DTPA: Diethylenetriaminepentaacetic acid
EDTA: Ethylenediaminetetraacetic acid
hMN-14: Labetuzumab
MES: M 2-(N-morpholino)ethanesulfonic acid
NIR: Near infrared
PBS: Phosphate-buffered saline
PDT: Photodynamic therapy
ROS: Reactive oxygen species
tPDT: Targeted photodynamic therapy
