# Supplementary material for: Carcinoembryonic antigen-targeted photodynamic therapy in colorectal cancer models
Source: EJNMMI Res. 2019 Dec 11;9:108. doi: 10.1186/s13550-019-0580-z (PMC6906275; doi:10.1186/s13550-019-0580-z)
Supplement: Supplementary file 1 — Additional file 1: Figure S2. Ex vivo biodistribution of two mice with subcutaneous LoVo tumors, one day after 111In-labeled DTPA-hMN-14-IRDye700DX injection. [file 13550_2019_580_MOESM1_ESM.docx]

**Carcinoembryonic antigen-targeted photodynamic therapy in colorectal cancer models**

*Fortuné M.K. Elekonawo^1^, Desirée L. Bos^1^, David M. Goldenberg^2,3^, Otto C. Boerman^1^, Mark Rijpkema^1^*

^1^ Department of Radiology and Nuclear Medicine, Radboud University Medical Center, Nijmegen, The Netherlands

Corresponding author e-mail: [Fortune.elekonawo@radboudumc.nl](mailto:Fortune.elekonawo@radboudumc.nl)

Supplementary Figure 2:


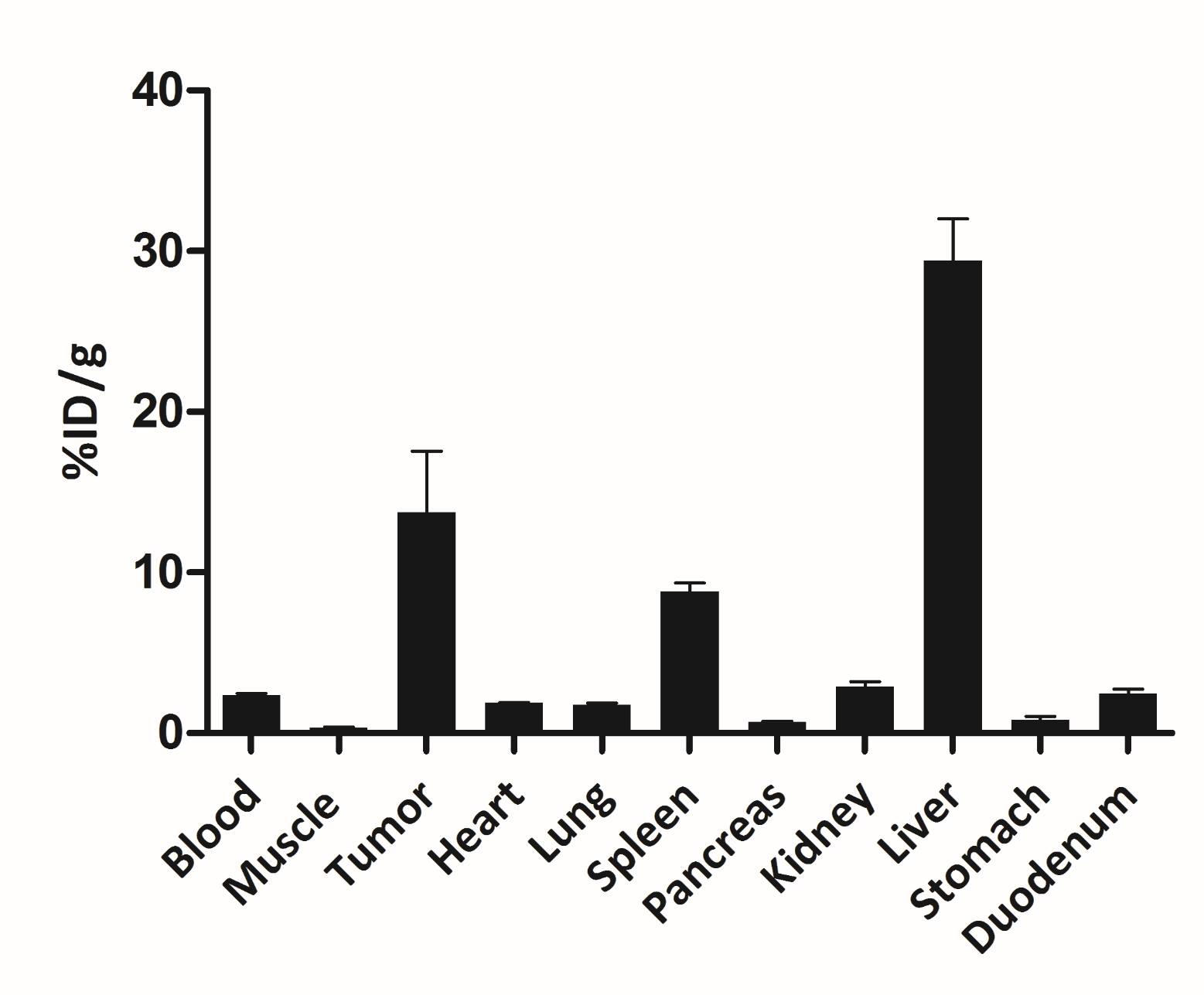


Supplementary Figure S2: *Ex vivo* biodistribution of two mice with subcutaneous LoVo tumors, one day after ^111^In-labeled DTPA-hMN-14-IRDye700DX injection.
