# Supplementary material for: Carcinoembryonic antigen-targeted photodynamic therapy in colorectal cancer models
Source: EJNMMI Res. 2019 Dec 11;9:108. doi: 10.1186/s13550-019-0580-z (PMC6906275; doi:10.1186/s13550-019-0580-z)
Supplement: Supplementary file 2 — Additional file 2: Figure S1. the CEA binding availability of the cell lines used in this study. Note the (arbitrary) classification in high (>5% specific binding) and low (<5% specific binding) binding of DTPA-hMN-14-IRDye700DX. [file 13550_2019_580_MOESM2_ESM.docx]

**Carcinoembryonic antigen-targeted photodynamic therapy in colorectal cancer models**

*Fortuné M.K. Elekonawo^1^, Desirée L. Bos^1^, David M. Goldenberg^2,3^, Otto C. Boerman^1^, Mark Rijpkema^1^*

^1^ Department of Radiology and Nuclear Medicine, Radboud University Medical Center, Nijmegen, The Netherlands

Corresponding author e-mail: [Fortune.elekonawo@radboudumc.nl](mailto:Fortune.elekonawo@radboudumc.nl)

Supplementary Figure 1:

**
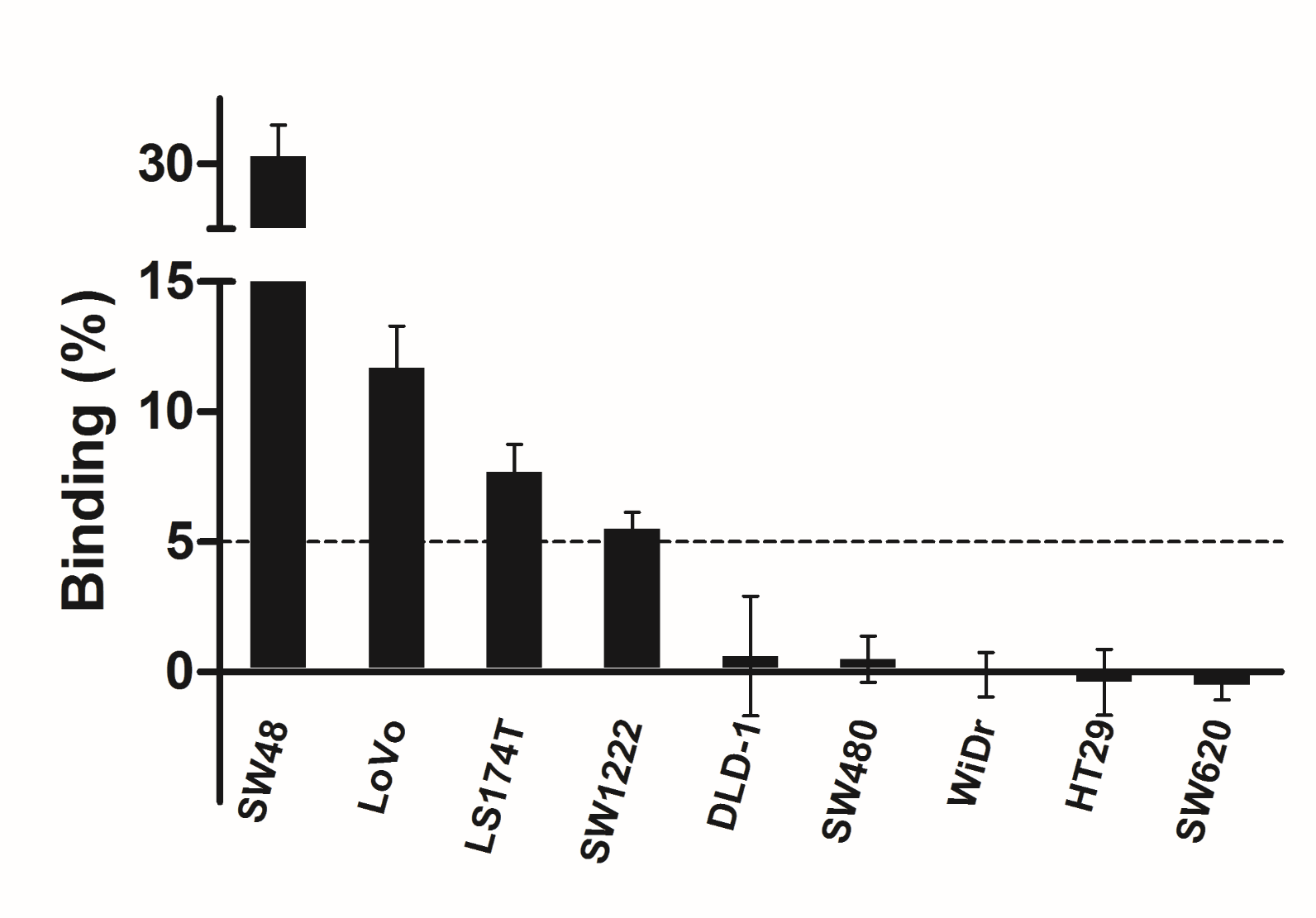
**

Supplementary Figure S1: the CEA binding availability of the cell lines used in this study. Note the (arbitrary) classification in high (>5% specific binding) and low (<5% specific binding) binding of DTPA-hMN-14-IRDye700DX.
